# An Optimization Algorithm for Computer-Aided Diagnosis of Breast Cancer Based on Support Vector Machine

**DOI:** 10.3389/fbioe.2021.698390

**Published:** 2021-07-05

**Authors:** Yifeng Dou, Wentao Meng

**Affiliations:** ^1^Network Information Center, Tianjin Baodi Hospital, Tianjin, China; ^2^Baodi Clinical College, Tianjin Medical University, Tianjin, China

**Keywords:** breast cancer, computer-aided diagnosis, support vector machine, optimization, machine learning, classification

## Abstract

As one of the most vulnerable cancers of women, the incidence rate of breast cancer in China is increasing at an annual rate of 3%, and the incidence is younger. Therefore, it is necessary to conduct research on the risk of breast cancer, including the cause of disease and the prediction of breast cancer risk based on historical data. Data based statistical learning is an important branch of modern computational intelligence technology. Using machine learning method to predict and judge unknown data provides a new idea for breast cancer diagnosis. In this paper, an improved optimization algorithm (GSP_SVM) is proposed by combining genetic algorithm, particle swarm optimization and simulated annealing with support vector machine algorithm. The results show that the classification accuracy, MCC, AUC and other indicators have reached a very high level. By comparing with other optimization algorithms, it can be seen that this method can provide effective support for decision-making of breast cancer auxiliary diagnosis, thus significantly improving the diagnosis efficiency of medical institutions. Finally, this paper also preliminarily explores the effect of applying this algorithm in detecting and classifying breast cancer in different periods, and discusses the application of this algorithm to multiple classifications by comparing it with other algorithms.

## Introduction

Health is the foundation of all-round development of human beings. The incidence rate of breast cancer worldwide has been increasing since the end of 1970s. Breast Cancer is a malignant tumor of abnormal breast cell division and proliferation. The incidence of breast cancer is more prominent in female patients. A United States survey shows that in 2016, 16,85,210 cases of new cancer and 595 cases of cancer were found. Among 690 cancer deaths, breast cancer is the main cause of cancer death in women aged 20–59 (Siegel et al., 2016). Each year, the number of new breast cancer cases and deaths in China account for 12.2 and 9.6% of the world’s total, respectively. In view of this serious social reality, there is an urgent need to carry out research on the risk of breast cancer, including the cause analysis and prediction of breast cancer risk diagnosis based on historical data (Li Y. et al., 2020). In the examination, the characteristics of cell size, shape, and mass thickness are considered as the criteria to distinguish benign from malignant tumors, while the characteristics of age, tumor size, menopause, number of lymph nodes involved and radiotherapy are considered as the factors influencing the recurrence of breast cancer. It is difficult for doctors to manually determine whether breast cancer is benign or not and the recurrence of breast cancer according to the complex characteristic data, but computer technology can analyze and predict the existing data.

Artificial intelligence (AI) is the product of the rapid development of computer technology. It has a profound impact on the development of human society and the progress of science and technology. At this stage, artificial intelligence has been widely used in clinic. With the development of technology and the availability of big data, the application and development of artificial intelligence in medical disease diagnosis has become a research hotspot in today’s era. As one of the important means in artificial intelligence, in 1959, Arthur Samuel proposed the concept of machine learning, that is, using algorithms to make machines learn from a large number of data, to obtain the method of new data analysis and research (Skoff, 2017).

At present, researchers have used deep learning or machine learning methods to study different breast cancer data. Khan et al. (2019) used the method of combining transfer learning and deep learning to detect and classify breast cancer cells, and achieved high accuracy. Abbass (2002) proposed a neural network method based on differential evolution algorithm and local search to predict breast cancer, and the standard deviation of its test accuracy is 0.459 lower than that of Fogel et al. (1995). Abdikenov et al. (2019) used the evolutionary algorithm NSGA III (non-dominated sorting genetic algorithm – III) to initialize the deep neural network and optimize its super parameters for the prognosis of breast cancer. Liu et al. (2019) proposed an end-to-end deep learning system combined with full convolution network to extract breast region data, and the results are highly correlated with the diagnosis made by pathologists. Lu et al. (2019) proposed a novel genetic algorithm based online gradient boosting (GAOGB) model to predict the diagnosis and prognosis of breast cancer in real time through online learning (Oza, 2005) technology. The above research shows that the application of artificial intelligence in the medical field is practical and effective. The application of existing machine learning methods in the medical field helps medical workers improve work efficiency and reduce work burden. People are trying to improve the traditional algorithm while applying computer technology to the medical field.

In this paper, Support Vector Machine (SVM) is taken as a breakthrough point. The choice of penalty parameter c and g in SVM kernel function is directly related to the effectiveness and accuracy of SVM algorithm in solving dichotomy. According to previous research methods, there are mainly 5 optimization methods for the above two important parameters, namely, empirical selection method, grid selection method, genetic optimization algorithm, particle swarm optimization algorithm, and ant colony optimization algorithm so on (Ali and Abdullah, 2020; Kouziokas, 2020; Li X. et al., 2020; Arya Azar et al., 2021; Ramkumar et al., 2021). Although these optimization algorithms have been applied to some extent and achieved some effects, they all have problems of different degrees. For example, the empirical selection method is highly experienced by users and highly dependent on samples, which lacks sufficient theoretical support. The disadvantage of grid selection method lies in the step size selection. If the step size selection is too large, it is easy to fall into the local optimum; if the step size selection is too small, the calculation amount will be too large. The genetic optimization algorithm needs to go through three steps of selection, crossover and mutation. The parameter setting is relatively complex, the convergence speed is slow, and it is easy to fall into the local optimal solution. Particle swarm optimization (PSO) SVM has the advantage of faster convergence speed and fewer parameters, but it is also easy to fall into local optimal. The combination of genetic or particle swarm optimization and simulated annealing (SA) to optimize SVM parameters improves the convergence speed and improves the poor local optimization ability to some extent. However, poor stability may occur in some practical applications. Therefore, how to use the advantages of three heuristic algorithms to optimize the selection of parameters in support vector machines, so that the algorithm to achieve the best classification performance is the focus of this paper.

## Conceptual Principle

### Support Vector Machine

Support vector machine was proposed by Vapnik (1995). The basic idea of the algorithm is to map the input data into a high-dimensional space through non-linear transformation and establish the optimal linear classification surface to classify the two sample categories correctly. Based on the principle of structural risk minimization, the SVM model is classified by calculating the optimal separating hyperplane (OSH) (Zhou et al., 2018). The larger the interval between the optimal hyperplanes, the stronger the generalization ability of the established SVM model. Suppose that the training sample set {(*x*_*i*_, *y*_*i*_), *i* = 1,2,…,l} with the size of 1, its data samples can only be divided into two categories. If it belongs to the first type of samples, it is recorded as positive (*y*_*i*_ = 1), otherwise it belongs to the second category and is recorded as a negative value (*y*_*i*_ = −1). At this time, we need to construct a discriminant function to make the function classify the test data samples as correctly as possible. If there is a classification hyperplane

(2-1) w⋅x+b=0

bring

(2-2) $$\left\{ \begin{array}{c} w\cdot x_{\text{i}}+b\geq 1,y_{\text{i}}=1\\ w\cdot x_{\text{i}}+b\leq -1,y_{\text{i}}=-1,i=1,2,\ldots ,l \end{array}\right.$$

We call the training sample set is linearly separable. *w*⋅*x* is called the inner product of vector *w* ∈ *R*^*N*^ and vector *x* ∈ *R*^*N*^, and *w* ∈ *R*^*N*^ and *b* ∈ *R* in formula (2-1) and formula (2-2) are normalized. For formula (2-2), it can be rewritten as follows:

(2-3) $$y_{\text{i}}\left(w\cdot x_{\text{i}}+b\right)\geq 1,i=1,2,\ldots ,l$$

According to the definition of the optimal hyperplane, the following discriminant functions can be obtained

(2-4) y(x)=sign(w⋅x+b)

Its generalization ability is the best, and sign (⋅) is the symbol function. The solution of the optimal hyperplane needs to maximize 2/||*w*||, that is to say it can be transformed into the following quadratic programming problem composed of objective function and constraint conditions

(2-5) $$\begin{array}{c} \min_{w,b}\frac{{||w||}^{2}}{2}\\ s.t.y_{\text{i}}\left(w\cdot x_{\text{i}}+b\right)\geq 1,i=1,2,\ldots ,l \end{array}$$

When the training sample set is linear and indivisible, it is necessary to introduce a non-negative parameter, i.e., relaxation variable **ξ**_*i*_, *i* = 1,2,…, *l*. at this time, the optimization problem of classification hyperplane is transformed into the form shown in formula (2-6).

(2-6) $$\begin{array}{c} \min_{w,b,\xi }\frac{{||w||}^{2}}{2}+c\sum_{i=1}^{l}\xi_{\text{i}}\\ s.t.\left\{ \begin{array}{c} y_{\text{i}}\left(w\cdot x_{\text{i}}+b\right)\geq 1-\xi_{\text{i}}\\ \xi_{\text{i}}\geq 0,i=1,2,\ldots ,l \end{array}\right. \end{array}$$

Where *c* is the constraint parameter, also known as the penalty parameter. The higher the value of *c*, the greater the penalty for error classification. Using Lagrange multiplier method to solve the problem

(2-7) $$\begin{array}{c} \begin{array}{c} \underset{\alpha ,\beta ,w,b,\xi }{\max\min}\{L_{\text{p}}=\frac{{||w||}^{2}}{2}+c\sum_{i=1}^{l}\xi_{\text{i}}-\sum_{i=1}^{l}\alpha_{\text{i}}[y_{\text{i}}\left(w\cdot x_{\text{i}}+b\right) \end{array}\\ \begin{array}{c} -1+\xi_{\text{i}}]-\sum_{i=1}^{l}\beta_{\text{i}}\xi_{\text{i}}\} \end{array}\\ s.t.\left\{ \begin{array}{c} \alpha_{\text{i}}\geq 0\\ \beta_{\text{i}}\geq 0 \end{array}\right. \end{array}$$

Where α_*i*_ and β_*i*_ are Lagrange multipliers

(2-8) $$\frac{\partial L_{\text{p}}}{\partial w}=0\to w=\sum_{i=1}^{l}\alpha_{\text{i}}y_{\text{i}}x_{\text{i}}$$

(2-9) $$\frac{\partial L_{\text{p}}}{\partial b}=0\to \sum_{i=1}^{l}\alpha_{\text{i}}y_{\text{i}}=0$$

(2-10) $$\frac{\partial L_{\text{p}}}{\partial \xi_{\text{i}}}=0\to c-\alpha_{\text{i}}-\beta_{\text{i}}=0$$

By substituting formula (2-8) to (2-10) into formula (2-7), the dual optimization problem form is obtained

(2-11) $$\begin{array}{c} \max_{\alpha }\left\{L_{D}=\sum_{i=1}^{l}\alpha_{\text{i}}-\frac{1}{2}\sum_{i=1}^{l}\sum_{j=1}^{l}\alpha_{\text{i}}\alpha_{\text{j}}y_{\text{i}}y_{\text{j}}x_{\text{i}}x_{\text{j}}\right\}\\ s.t.\left\{ \begin{array}{c} 0\leq \alpha_{\text{i}}\leq c\\ \sum_{i=1}^{l}\alpha_{\text{i}}y_{\text{i}}=0 \end{array}\right. \end{array}$$

The α_*i*_ obtained by optimization may be (a) α_*i*_ = 0; (b) 0 < α_*i*_ < *c*; (c) α_*i*_ = *c*. According to formula (2-8), only when the support vector has a positive effect on the optimal hyperplane and discriminant function, the corresponding learning method is called support vector machine algorithm. In support vector, *x*_*i*_ corresponding to c is called boundary support vector (BSV), which is actually the training sample points that are misclassified; (b) The corresponding *x*_*i*_ is called normal support vector (NSV). According to Karush–Kuhn–Tucher condition (Chauhan and Ghosh, 2021), the product between Lagrange multiplier and corresponding constraint is equal to 0 when the sample point is optimal

(2-12) $$\left\{ \begin{array}{c} \alpha_{\text{i}}\left[y_{\text{i}}\left(w\cdot x_{\text{i}}+b\right)-1+\xi_{\text{i}}\right]=0\\ \beta_{\text{i}}\xi_{\text{i}}=0 \end{array}\right.$$

For the standard support vector (0 < α_*i*_ < *c*), β_*i*_ > 0 is obtained from formula (2-10). Therefore, β_*i*_ = 0 can be obtained from formula (2-12). Therefore, it can be seen that all the criteria satisfy the following requirements for any standard support vector *x*_*i*_,

(2-13) $$y_{\text{i}}\left(w\cdot x_{\text{i}}+b\right)=1$$

the parameter *b* is calculated

(2-14) $$b=y_{\text{i}}-w\cdot x_{\text{i}}=y_{\text{i}}-\sum_{x_{\text{i}}\in NSV,x_{\text{j}}\in SV}\alpha_{\text{j}}y_{\text{j}}x_{\text{j}}x_{\text{i}}$$

The value of *b* is calculated for all standard support vectors, and then the average value of the results is obtained

(2-15) $$b=\frac{1}{N_{NSV}}\sum_{x_{\text{i}}\in NSV}\left(y_{\text{i}}-\sum_{x_{\text{i}}\in SV}\alpha_{\text{j}}y_{\text{j}}x_{\text{j}}x_{\text{i}}\right)$$

Where *N*_*NSV*_ is the number of the standard support vectors. According to formula (2-13), the support vector machine model is the sample data that meets the requirements of formula (2-3).

### Kernel Function Selection for Support Vector Machine Algorithm

The use of support vector machines to solve pattern classification problems usually requires the selection of an appropriate kernel function. Since the low-dimensional space vector sample set is usually difficult to divide, we usually use to map the low-dimensional space vector sample set into the high-dimensional feature space, but the consequent problem is to increase the computational complexity, and the emergence of the kernel function is a good solution to the problem. Theoretically, any function that can satisfy the Merce condition can be used as the kernel function of a support vector machine algorithm, but the different choices of kernel functions can lead to different algorithms and directly lead to different performance of their classifiers. Therefore, the selection of the appropriate kernel function is crucial to effectively improve the distribution of feature vectors in the high-dimensional feature space, thus making the structure of the classifier simpler; at the same time, even if a certain kernel function is selected, the selection of the corresponding parameters in the kernel function, such as the order in the polynomial kernel function and the width parameter in the Gaussian kernel function, also needs to be deliberated.

The most studied kernel functions are mainly of the following types, one is linear kernel function, as shown in formula (2-16), which mainly solves linear classification problems.

(2-16) $$K\left(x_{\text{i}},x_{\text{j}}\right)=x_{\text{i}}\cdot x_{\text{j}}$$

Second, the polynomial kernel function, as shown in formula (2-17), is obtained as a polynomial classifier of order *q*.

(2-17) $$K\left(x_{\text{i}},x\right)={\left(x_{\text{i}}\cdot x+1\right)}^{d}$$

Third, the radial basis function (referred to as the RBF kernel function), as shown in formula (2-18).

(2-18) $$K\left(x,x_{\text{i}}\right)=\exp\left(-\frac{{||{\text{x-x}}_{\text{i}}||}^{2}}{\sigma^{2}}\right)$$

The resulting classifier differs from the traditional RBF method in that it has a support vector corresponding to the center of each basis function, where the weights of the output are determined automatically by the algorithm. A Sigmoid function can also be used as the inner product, i.e.,

(2-19) $$K\left(x,x_{\text{i}}\right)=\tanh\left(v\left(x\cdot x_{\text{i}}\right)+c\right)$$

The support vector machine algorithm implemented in this case is equivalent to a multilayer perceptron network with hidden layers, in which the number of hidden layer nodes is also determined automatically by the algorithm, and it is also able to better solve the problem of local minima in neural networks. Based on this, and also considering that the SVM algorithm is not sensitive to the selection of the kernel, this paper uses the radial basis kernel function, which is also called Gaussian Kernel. The classification accuracy factor σ in the RBF kernel is the parameter that needs to be adjusted, and the different values of σ will also have a great impact on the nature of the classifier and the correct recognition rate, etc.

### Optimization Algorithm

For the improvement of local optimization and global optimization, this paper uses the genetic algorithm and particle swarm optimization algorithm in the algorithm to determine the respective population optimal solution, so as to seek the global optimal solution as the parameter input of SVM, so as to achieve a good balance between global and local search optimization. Through the assignment between the optimal particle and the worst chromosome or between the worst particle and the optimal chromosome, the two search algorithms complement each other and accelerate the convergence speed of the algorithm.

For the improvement that particle swarm optimization algorithm is easy to fall into local optimum, this paper takes into account the role of inertia factor ω in particle velocity and position update in formula (2-20), Because ω reflects the ability of particles to inherit the previous velocity, when the value is large, the particle swarm optimization has strong search ability in the early stage, but it is not conducive to ensure the optimal solution when the search enters the late stage; When the value of ω is small, the effect is just the opposite. When the value is small, the search ability of particle swarm optimization is enhanced, but the ability of global search for optimal solution is decreased. Therefore, in order to improve this deficiency, the harmonic inertia factor is adopted, as shown in formula (2-22).

(2-20) $$v_{i,j}\left(t+1\right)=\omega v_{i,j}\left(t\right)+c_{1}r_{1}\left[p_{i,j}-x_{i,j}\left(t\right)\right]+c_{2}r_{2}\left[p_{g,j}-x_{i,j}\left(t\right)\right]$$

(2-21) $$x_{i,j}\left(t+1\right)=x_{i,j}\left(t\right)+v_{i,j}\left(t+1\right)$$

The meanings of parameters in the above two formulas are as follows:

ω represents the inertia weight of particles, and *c*_1_ and *c*_2_ represent the self-learning factor and global learning factor of particles, respectively. *r*_1_ and *r*_2_ represent random numbers between [0–1]. In order to make particles search in effective space, it is generally necessary to limit the search space of particles, that is to limit the position to [*x*_*min*_, *x*_*max*_]. At the same speed, a range [*v*_*min*_, *v*_*max*_] should be set instead of blindly optimizing. This setting can control the movement of particles.

(2-22) $$\omega_{\text{m}}=\omega_{1}-\frac{\left(\omega_{1}-\omega_{2}\right){\left(m-1\right)}^{2}}{t^{2}}$$

Where *m* is the number of iterations and *t* is the maximum evolution algebra.

In order to improve the local search ability of particle swarm optimization (PSO), a simulated annealing algorithm is introduced in this paper. Metropolis criterion (Wang et al., 2019) is used to determine whether to accept the new location of particles, suppose that the change of fitness of the particle in the new position is Δ*f*, if Δ*f* ≥ 0, then accept the new position of the particle at time t;If Δ*f* < 0, the acceptance probability is calculated according to formula (2-23). By comparing with the threshold value, it is a standard normal distribution random quantity with a mean value of 0 and a standard deviation of 1. When *P*_a_ > *P* the bad position is accepted.

(2-23) $$P_{\text{a}}=\exp\left(\frac{\Delta f}{t}\right),t=KT$$

Where *t* is the control parameter, *K* is the Boltzmann constant in physics, and *T* is the temperature of the material.

**FIGURE 1:**
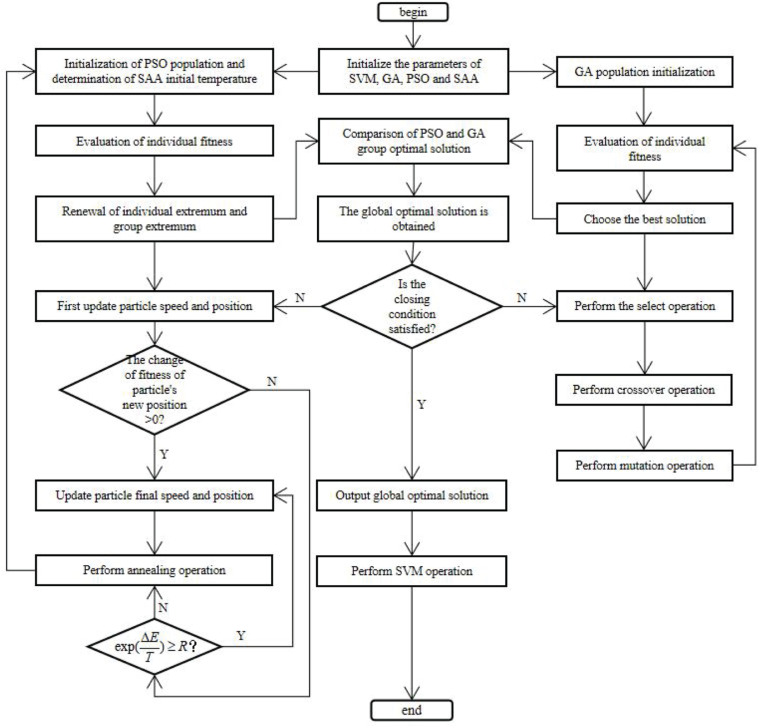
Flow chart of GSP_SVM algorithm.

Both genetic algorithm and particle swarm optimization belong to the branch of evolutionary algorithm. Both of them are suitable for solving discrete problems, especially 0–1 non-linear optimization, integer programming and mixed integer programming. Therefore, this paper selects GA and PSO as basic algorithms. At the same time, in order to make full use of the local search solution space of GA and the fast convergence ability of PSO algorithm, and to improve the poor local search ability of PSO algorithm in the later stage, the simulated annealing algorithm is introduced f or optimization. In this paper, a new algorithm combining three classical algorithms to optimize support vector machine (GSP_SVM) is proposed. By comparing the population optimal solutions obtained from GA and PSO algorithm, the overall optimal solution is found. In this paper, the accuracy of training classification is taken as fitness value. If the fitness of PSO optimal solution is higher than that of GA, it is regarded as the global optimal solution and assigned to the worst chromosome in GA. however, if the fitness of PSO optimal solution is lower than that of GA, the chromosome with the highest fitness is regarded as the global optimal solution and assigned to the particle with the worst fitness, and then iterative calculation is carried out until the algorithm is implemented Termination. The overall framework of the algorithm is shown in Figure 1.

## Experiment

### Data Set

In order to verify the effectiveness and feasibility of the gsposvm algorithm proposed in this paper, we use the breast cancer data set^1^ provided by Dr. William H. wolberg of the Wisconsin Medical School in the United States. Each data sample in the medical data set has 10 attribute variables, which are case code number, tumor thickness value, cell size uniformity, cell shape uniformity, edge viscosity, single epithelial cell size, naked nucleus, boring chromosome, normal nucleus and mitotic number. Except the case code number, the values of the other 9 attributes were all [1,10]. The binary variable was to judge the characteristics of breast cancer. 1 was malignant and 2 was benign. In order to get a better prediction effect, this section makes a study on the original data set of “breast cancer”- wisconsin.data to preserve the authenticity of the data, the redundant attributes are removed 16 data samples were eliminated, and the final experimental data samples were 683. Finally, in order to reduce the value range of some attributes which are too large while others are too small, so that the large number will submerge the decimal. At the same time, in order to avoid the difficulties in numerical calculation due to the calculation of kernel function, the data of training set and test set are normalized, and the data is scaled to [0,1].

### Evaluating Indicators

In order to better explain the evaluation index used in this paper, we first give a confusion matrix about binary classification problem, as shown in Table 1.

**TABLE 1:** Contingency table for binary classification problems.

Actual *Class*_*i*_	Prediction	
	Judged as *Class*_*i*_	Not *Class*_*i*_
The record belongs to *Class*_*i*_	*True Positive* (*TP*_*i*_)	*False Negative* (*FN*_*i*_)
The record does not belongs to *Class*_*i*_	*False Positive* (*FP*_*i*_)	*True Negative* (*TN*_*i*_)

Based on the Precision and recall rate, the receiver characteristic curve, namely AUC, F-measure, total accuracy G are used to evaluate the application effect of the proposed optimization algorithm in unbalanced data sets.

Precision: refers to the ratio of the number of records that the classifier can correctly determine as the category and the total number of records that should be determined as the category. As shown in formula (3-1), the precision rate represents the classification accuracy of the classifier itself. If the *TP*_*i*_ is larger and the *FP*_*i*_ is smaller, the precision value will be larger, which means that the probability of the classifier’s misclassification on this category will be smaller.

(3-1) $$Precision=\frac{TP_{i}}{TP_{\text{i}}+FP_{\text{i}}}\times 100\%$$

Recall: refers to the ratio of the number of records that can be correctly determined by the classifier to the total number of records in the classification records that should be the category. As shown in formula (3-2), recall reflects the completeness of the classification results of the classifier. If the greater the *TP*_*i*_ is, the smaller the *FN*_*i*_ is, the greater the recall value is, which means that the fewer records should have been missed by the classification system.

(3-2) $$Recall=\frac{TP_{\text{i}}}{TP_{\text{i}}+FN_{\text{i}}}\times 100\%$$

Sensitivity: the proportion of correct number of multi class discrimination in all multi class samples, and the calculation method is consistent with the calculation formula of recall rate.

Specificity: the proportion of the correct number of minority discrimination in all minority samples. The calculation method is shown in formula (3-3).

(3-3) $$Specificity=\frac{TN_{\text{i}}}{TN_{\text{i}}+FP_{\text{i}}}\times 100\%$$

Total accuracy *G*: considering the classification performance of minority and majority records, the calculation method is the geometric average of specificity and sensitivity. It can be seen from formula (3-4) for details. Therefore, *G* is also called geometric average, and the accuracy increases monotonically with the values of specificity and sensitivity in [0,1].

(3-4) $$G=\sqrt{Specificity*Sensitivity}$$

*F*_β_: considering the difference between precision rate and recall rate, the formula is as follows:

(3-5) $$F_{\beta }=\frac{\left(\beta^{2}+1\right)\times Precision\times Recall}{\beta^{2}\times Precision+Recall}$$

(3-6) $$\begin{array}{c} F-measure=\frac{2*Precision*Recall}{Precision+Recall}\\ =\frac{2*Precision*Sensitivity}{Precision+Sensitivity} \end{array}$$

The *F*_β_ measure value represents the trade-off between accuracy and recall when evaluating the performance of classifiers. β is used to adjust the proportion of precision and recall in the formula. Usually, when it is used in practice, it is taken as β = 1 to get the performance evaluation index *F*-measure of our common classifier. The calculation formula is as follows (3-6). F-measure is the harmonic mean of precision and recall. When the accuracy and recall are both high, the *F*-measure value will also increase. This index takes into account the recall and precision of minority records. Therefore, any change of any value can affect the size of *F*-measure. Therefore, it can show the classification effect of the classifier on the majority class and minority class, but it focuses on the classification effect of minority records is also discussed.

MCC: Matthew’s correlation coeffcient (3-7):

(3-7) $$MCC=\frac{TP\times TN-FP\times FN}{\sqrt{\left(TP+FN\right)\left(TP+FP\right)\left(TN+FP\right)\left(TN+FN\right)}}$$

AUC (area under the ROC curve): the area under the ROC curve, between 0.1 and 1. It can quantify the ROC curve and present the algorithm performance more intuitively. The larger the value is better. The larger the value is, the more likely the positive samples will be placed before the negative samples, so as to better classify.

### Results

#### Discussion of the Binary Classification Problems

In this paper, we use radial basis function, which is also known as Gaussian kernel function. The classification accuracy factor in RBF kernel is a parameter σ that needs to be adjusted. Different σ values will have a great impact on the properties of classifier and recognition accuracy. In this paper, a heuristic search method is used to find the optimal parameters in the model selection, so as to achieve the optimal performance for the classification and prediction.

In order to verify the effect of different optimization algorithms on the optimization of support vector machine parameters, this paper uses several algorithms for experimental comparison: (1) Based on the most original support vector machine algorithm; (2) Based on principal component analysis support vector machine algorithm (PCA_SVM) (Tao and Cuicui, 2020); (3) Support vector machine algorithm based on grid search optimization (GS_SVM) (Fayed and Atiya, 2019); (4) Support vector machine algorithm based on genetic algorithm optimization (GA_SVM) (Guan et al., 2021); (5) Particle swarm optimization based support vector machine algorithm (PSO_SVM) (Zhang and Su, 2020), in order to compare the genetic algorithm, particle swarm optimization algorithm and simulated annealing algorithm based on the fusion algorithm to optimize the parameters of support vector machine (GSP_SVM).

For better performance comparison and algorithm verification, we randomly take 50, 60, 70, and 80% of the data as labeled data and training data, and the remaining data as unlabeled sample data and test sample set. In order to balance the random effect, the average value of 10 repeated independent running results is used for the reported experimental results. The specific results are shown in Table 2.

**TABLE 2:** Experimental results.

Evaluating indicator	Proportion of training data	SVM	PCA_SVM	GA_SVM	GS_SVM	PSO_SVM	GSP_SVM
Precision	50%	0.9853	0.9417	0.9906	0.9804	0.9450	0.9716
	60%	0.9695	0.9519	0.9708	0.9711	0.9586	**0.9818**
	70%	0.9841	0.9697	0.9853	0.9924	**0.9927**	0.9699
	80%	0.9878	0.9667	0.9865	0.9667	0.9778	**1.0000**
	90%	0.9773	0.9524	0.9722	0.9778	0.9778	**1.0000**
Recall	50%	0.9526	0.9713	0.9251	0.9524	**0.9810**	0.9716
	60%	0.9578	0.9700	0.9595	0.9711	0.9701	**0.9759**
	70%	0.9612	0.9771	0.9710	0.9489	0.9577	**0.9847**
	80%	0.9529	0.9667	0.9359	**0.9886**	0.9670	0.9655
	90%	0.9556	0.9756	0.9722	0.9565	**0.9778**	0.9762
G	50%	1.0009	0.9061	**1.0214**	0.9934	0.9172	0.9677
	60%	0.9740	0.9112	0.9698	0.9640	0.9483	**0.9839**
	70%	0.9928	0.9562	0.9841	**1.0112**	1.0053	0.9525
	80%	1.0039	0.9508	1.0159	0.9429	0.9717	**1.0177**
	90%	0.9785	0.9374	0.9825	0.9760	0.9673	**1.0121**
*F*-measure	50%	0.9687	0.9611	0.9567	0.9662	0.9626	**0.9716**
	60%	0.9636	0.9648	0.9651	0.9711	0.9643	**0.9789**
	70%	0.9725	0.9734	**0.9781**	0.9701	0.9749	0.9773
	80%	0.9701	0.9667	0.9605	0.9775	0.9724	**0.9825**
	90%	0.9663	0.9639	0.9722	0.9670	0.9778	**0.9880**
MCC	50%	0.9209	0.8933	0.8828	0.9146	0.9008	**0.9254**
	60%	0.9082	0.8921	0.9058	0.9211	0.9073	**0.9463**
	70%	0.9273	0.9252	0.9336	0.9150	0.9222	**0.9358**
	80%	0.9226	0.9014	0.9122	0.9355	0.9176	**0.9538**
	90%	0.9030	0.9077	0.9410	0.9009	0.9343	**0.9696**
AUC	50%	0.9707	0.9652	0.9561	0.9695	0.9681	**0.9708**
	60%	0.9592	0.9761	0.9688	0.9705	0.9652	**0.9865**
	70%	0.9737	0.9648	0.9758	0.9573	0.9752	**0.9782**
	80%	0.9812	0.9708	0.9651	0.9711	0.9731	**0.9864**
	90%	0.9729	0.9512	0.9679	0.9605	0.9720	**0.9890**

*Bolded values represent the optimal values obtained by the algorithm for the evaluation metrics on this training set.*

Since the parameters of SVM and PCA_SVM algorithms are set to fixed values, *c* is 100 and *g* is 4, all the other algorithms are optimized for SVM parameters except these two algorithms. On the whole, with the increase of training sample data, most of the values of the evaluation matrix have a positive growth trend. It can be seen from the above table that among all the optimization algorithms, the support vector machine algorithm (GSP_SVM) based on the fusion of three classical optimization algorithms has improved the value of each evaluation index to varying degrees compared with other algorithms. The accuracy rate, recall rate, sensitivity, *F*-measure measurement value and other four evaluation indicators are presented in 60, 70, and 80% training data, respectively, the best result, in AUC, the best is in 70 and 80% training data.

This paper also investigates the accuracy, the most basic evaluation index of classification algorithm. The calculation method is shown in formula (3-8). No matter which category, as long as the prediction is correct, the number is placed on the numerator, and the denominator is the number of all the data. It shows that the accuracy is the judgment of all the data, and it is the evaluation index that can directly reflect the advantages and disadvantages of the algorithm. The accuracy of each algorithm on the training data set with different proportions can be seen from Table 3.

(3-8) $$Accuracy=\frac{TP+TN}{TP+TN+FP+FN}$$

**TABLE 3:** The experimental results of classification accuracy of algorithms.

	SVM	PCA_SVM	GA_SVM	GS_SVM	PSO_SVM	GSP_SVM
50%	0.9619	0.9501	0.9443	0.9589	0.9531	0.9648
60%	0.9560	0.9524	0.9560	0.9634	0.9560	**0.9744**
70%	0.9657	0.9657	**0.9706**	0.9608	0.9657	**0.9706**
80%	0.9630	0.9559	0.9559	0.9706	0.9632	**0.9779**
90%	0.9559	0.9559	0.9706	0.9559	0.9706	**0.9853**
Avg	0.9605	0.9560	0.9595	0.9619	0.9617	**0.9746**

*Bolded values represent the optimal values obtained by the algorithm for the evaluation metrics on this training set.*

According to the above table, the results of the experiment on 50, 60, 80, and 90% training proportion data sets are the best, especially in the 90%, the training proportion data set reaches 0.9853. Therefore, combined with the experimental results of evaluation indexes in Table 2, it can be concluded that the algorithm proposed in this paper can obtain the optimal parameter values and classify more accurately.

#### Discussion of the Multiclass Problems

In the above study, we considered the effectiveness of the algorithm for evaluation on the dichotomous classification problem, and next, in this paper, we will initially explore the classification of the algorithm on the multiclassification problem. We use the dataset proposed by M. Zwitter and M. Soklic from the Institute of Oncology, University of Ljubljana, Yugoslavia (Bennett et al., 2002), which has 286 instances, each containing 10 attributes such as tumor size, number of invaded lymph nodes, presence or absence of nodal adventitious, mass location, etc., all of which are of enumerated type, according to which we ask experts to manually annotate The defective instances accounted for only 0.3% of the total data set, so they were directly discarded. A total of 277 instances consisting of 10 independent variables and 1 multicategorical variable were finally used for the experiment.

For the binary classification problem, we have many evaluation metrics because there are only two types of positive and negative classes, but they are not applicable for the multi-classification problem. In this paper, we choose the following evaluation metrics for the multi-classification problem: (1) Accuracy_score, which is the ratio of the total number of correctly classified data in the classification result. (2) Precision_score, i.e., the proportion of positive cases in the prediction results. (3) Recall_score, i.e., the proportion of true positive cases that are finally predicted to be positive. (4) F1_score, as a combination of accuracy and recall, is often used as a metric for multi-classification model selection. (5) Hamming_loss, which is a measure of the distance between the predicted label and the true label, takes a value between 0 and 1, the smaller the value the better, and a distance of 0 indicates that the predicted result is exactly the same as the true result. (6) Cohen_kappa_score, the value range is [0,1], the higher the value of this coefficient, the higher the accuracy of the classification achieved by the model. It is calculated as *k* = (*P*_o_ − *P*_e_)/(1 − *P*_e_), Where *P*_*o*_ denotes the overall classification accuracy and *P*_*e*_ denotes SUM (the number of true samples in class *i* ^∗^ number of samples predicted in class *i*)/total number of samples squared. (7) Jaccard_score, which is used to compare the similarity and difference between the true and predicted values. The larger the coefficient value, the higher the sample similarity, indicating the more accurate prediction.

The experiments in this section are in the form of ten-fold cross-validation, and the average of five experiments is calculated as the indicator results, where for the average price indicators (2)–(4) a micro-averaging approach is used, i.e., a global confusion matrix is established for each instance in the dataset without categorizing the statistics, and then the corresponding indicators are calculated. The experimental results are shown in Table 4.

**TABLE 4:** Evaluation results of multicategorical indicators.

Evaluating indicator	Proportion of training data	SVM	PCA_SVM	GA_SVM	GS_SVM	PSO_SVM	GSP_SVM
Accuracy_score	50%	0.8768	0.8261	0.8551	0.8478	0.8261	0.8957
	60%	0.9455	0.9273	0.9364	0.8455	0.8273	**0.9527**
	70%	0.9518	**0.9639**	**0.9639**	0.9157	0.8193	**0.9639**
	80%	**0.9636**	**0.9636**	0.9455	0.8909	0.9455	**0.9636**
	90%	0.9630	0.9630	0.9630	0.9630	0.8889	**0.9852**
Precision_score	50%	0.8768	0.8261	0.8551	0.8478	0.8261	**0.8957**
	60%	0.9455	0.9273	0.9364	0.8455	0.8273	**0.9527**
	70%	0.9518	**0.9639**	**0.9639**	0.9157	0.8193	**0.9639**
	80%	**0.9636**	**0.9636**	0.9455	0.8909	0.9455	**0.9636**
	90%	0.9630	0.9630	0.9630	0.9630	0.8889	**0.9852**
Recall_score	50%	0.8768	0.8261	0.8551	0.8478	0.8261	**0.8957**
	60%	0.9455	0.9273	0.9364	0.8455	0.8273	**0.9527**
	70%	0.9518	**0.9639**	**0.9639**	0.9157	0.8193	**0.9639**
	80%	**0.9636**	**0.9636**	0.9455	0.8909	0.9455	**0.9636**
	90%	0.9630	0.9630	0.9630	0.9630	0.8889	**0.9852**
F1_score	50%	0.8768	0.8261	0.8551	0.8478	0.8261	**0.8957**
	60%	0.9455	0.9273	0.9364	0.8455	0.8273	**0.9527**
	70%	0.9518	**0.9639**	**0.9639**	0.9157	0.8193	**0.9639**
	80%	**0.9636**	**0.9636**	0.9455	0.8909	0.9455	**0.9636**
	90%	0.9630	0.9630	0.9630	0.9630	0.8889	**0.9852**
Hamming_loss↓	50%	0.1232	0.1739	0.1449	0.1522	0.1739	**0.1043**
	60%	0.0545	0.0727	0.0636	0.1545	0.1727	**0.0473**
	70%	0.0482	0.0361	0.0361	0.0843	0.1807	**0.0361**
	80%	0.0482	0.0361	0.0361	0.0843	0.1807	**0.0361**
	90%	0.0370	0.0370	0.0370	0.0370	0.1111	**0.0148**
Cohen_kappa_score	50%	0.8053	0.7170	0.7647	0.7533	0.7205	**0.8313**
	60%	0.9132	0.8896	0.8952	0.7553	0.7149	**0.9255**
	70%	0.9224	0.9412	0.9397	0.8602	0.7107	**0.9434**
	80%	0.9403	0.9369	0.9142	0.8291	0.9127	**0.9428**
	90%	0.9444	0.9429	0.9330	0.9363	0.8273	**0.9764**
Jaccard_score	50%	0.7899	0.7108	0.7452	0.7358	0.7006	**0.8145**
	60%	0.8966	0.8648	0.8809	0.7264	0.6987	**0.9111**
	70%	0.9357	0.9293	**0.9359**	0.8478	0.6901	0.9348
	80%	0.9300	**0.9386**	0.8961	0.8110	0.8978	0.9340
	90%	0.9444	0.9383	0.9288	0.9290	0.8008	**0.9716**

*Bolded values represent the optimal values obtained by the algorithm for the evaluation metrics on this training set.*

## Discussion

### Discussion of the Binary Classification Problems

**FIGURE 2:**
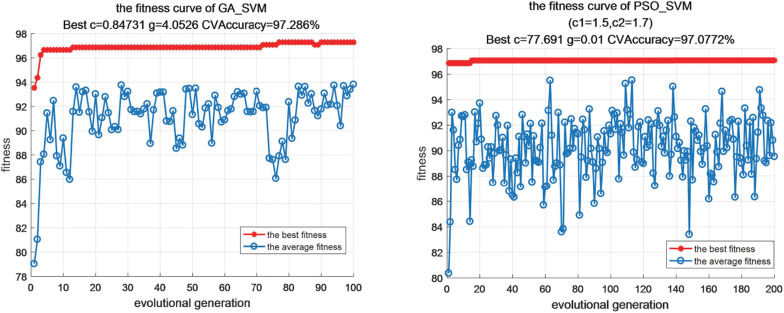
The visualization of iterative optimization.

In this experiments, we all assume that the range of penalty factor c is [0.1, 100], which is mainly used to control the tradeoff between model complexity and approximation error of classification model. If the penalty factor c is larger, the better the fitting degree of the algorithm is, but at the same time, the generalization ability of the algorithm will be reduced, which is not conducive to the popularization and application of the algorithm. At the same time, we also assume that the value range of parameter g in the selected Gaussian kernel function is [0.01,1000], which determines the classification accuracy of the algorithm. Through parameter optimization, we get the optimal parameter values of each algorithm, as shown in Table 5. Figure 2 also shows the visualization of iterative optimization of parameters c and g based on the algorithm of optimizing support vector machine parameters based on GA_SVM and PSO_SVM.

**TABLE 5:** The optimal parameters of algorithms.

Parameters	GA_SVM	GS_SVM	PSO_SVM	GSP_SVM
bestc	0.84731	0.0625	77.691	0.1
bestg	4.0526	0.7579	0.01	1.0164

From the above analysis, this paper uses the advantages of GA, PSO and SAA to improve the parameter optimization algorithm of support vector machine, which can balance the difference between global search optimization and local search optimization. Through the mutual assignment between the optimal particle and the worst chromosome or between the worst particle and the optimal dye, the genetic algorithm and particle swarm optimization algorithm complement each other. In the later stage, the local search ability of the sub group algorithm is insufficient, and the simulated annealing method is used to enhance it, the Metropolis criterion is used to select whether to accept new particles. According to the experimental results, we can also see that the improved SVM optimization algorithm can show good performance in the case of small samples and non-linear, and its robustness is high, the generalization ability is strong, and there is no problem of under fitting and over fitting.

### Extension to Multiclass Problems

**FIGURE 3:**
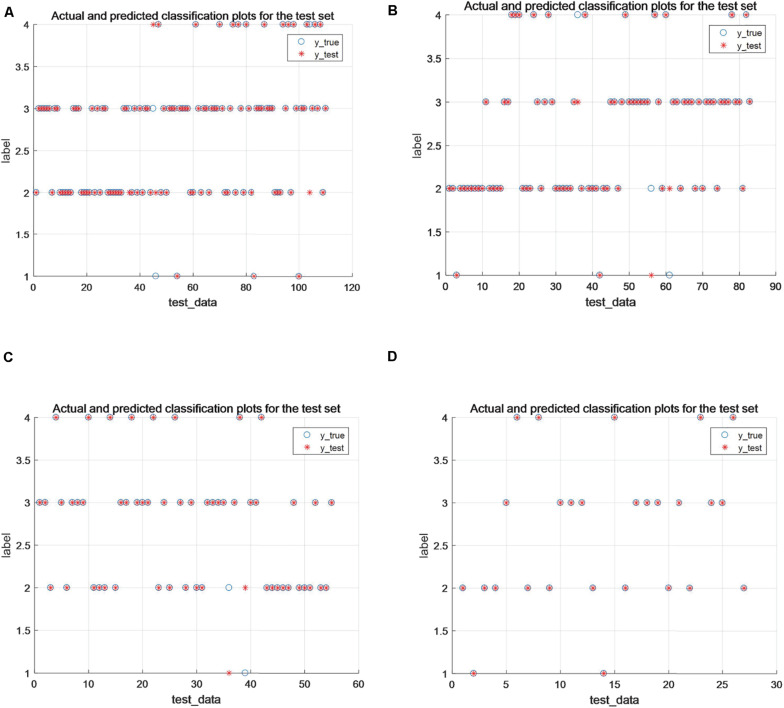
**(A–D)** Classification results under 60, 70, 80, and 90% training percentages, respectively.

From Table 4, it can be seen that, overall, the performance of this paper’s algorithm is optimal compared with other algorithms on data with 50, 60, and 90% training share, and the algorithm of this paper applied on 70% of the training dataset is consistent with Accuracy_score, Precision_score, Recall_score, and F1_score metrics with PCA_SVM and GA_SVM exhibit consistent results with SVM and PCA_SVM on 80% of the training set datasets. As the most commonly used metrics in evaluating multi-classification problems, the smaller the value of Hamming_loss, the closer the predicted label is to the true label, and the higher the value of Cohen_kappa_score, the better the classification accuracy of the algorithm. The algorithm in this paper shows optimal results in both metrics, especially in the 90% training data share, the Hamming_loss decreases to 0.0148 and Cohen_kappa_score reaches 97.64%, and Figure 3 shows the classification results of the algorithm in this paper on different training sets. Therefore, the algorithm proposed in this paper can also show better classification results when extended to multi-classification problems.

## Conclusion

In this paper, through the simultaneous interpretation of traditional optimization algorithms and machine learning methods, an algorithm combining three classical algorithms for optimization of support vector machines is proposed and trained and tested based on different breast cancer datasets, and the experimentally obtained classification accuracy, MCC, AUC, and other indexes reach high levels on the binary dataset. On the multiclassification dataset, the experimentally obtained metrics such as Hamming_loss minimum, Cohen_kappa_score and classification accuracy are optimal, which fully demonstrate that the method can provide decision support for breast cancer assisted diagnosis and thus significantly improve the diagnostic efficiency of medical institutions. Our research work will be based on this and will be developed into a breast cancer diagnosis recognition system, using artificial intelligence methods and combined with computer visualization to provide an auxiliary diagnostic basis for clinicians’ decision making through a graphical interface.

From the perspective of medical risk, in order to maximize the accuracy of the classification of malignant tumors, further research can be done on the combination of more complex kernel functions for different classifications. At the same time, medical institutions need to collect typical sample data purposefully to prevent the serious asymmetry of the two types of sample data. Of course, if we want to comprehensively improve the level of computer-aided diagnosis of diseases in medical institutions, we need to do further research on other high-risk diseases.

## Data Availability Statement

The original contributions generated for this study are included in the article/Supplementary Material, further inquiries can be directed to the corresponding author.

## Author Contributions

YD and WM conceived and designed the experimental protocol. YD involved in the analysis and designed the model of improved SVM. WM performed operations. YD wrote the first draft of the manuscript. WM reviewed and revised the manuscript. Both authors read and approved the final manuscript.

## Conflict of Interest

The authors declare that the research was conducted in the absence of any commercial or financial relationships that could be construed as a potential conflict of interest.

## Footnotes

1 http://www.csie.ntu.edu.tw/~cjlin/libsvmtools/datasets/binary.html#breast-cancer
